# Leech management before application on patient: a nationwide survey of practices in French university hospitals

**DOI:** 10.1186/s13756-018-0311-7

**Published:** 2018-02-05

**Authors:** Delphine Grau, Raphaël Masson, Maxime Villiet, Brigitte Lamy, French Hospitals Group, Nathalie Pelloquin, Christine Fagnoni, Arnaud Venet, Lucile Safrano, Claudine Hecquard, Christelle Prudent, Gaëlle Dunoyer, Marion Nouvel, Charleric Bornet, Grégory Rondelot, André Giesenfeld, Jacqueline Césari, Jean-François Huon, Ian Soulairol, Morgane Bonnet, Laetitia Grasset, Marie Desplechain, Sébastien Bauer, Voa Ratsimbazafy, Mireille Arfeuille, Aude Coquard, Dominique Paya, Valérie Sautou, Florence Lieutier, Isabelle Hermelin, Ludmilla Tatem, Vincent Gicquel, Delphine Merger

**Affiliations:** 1 Department of Clinical Pharmacy, CHU de Montpellier, 371, Avenue du Doyen Gaston Giraud, 34090 Montpellier, France; 2 UMR 5569 HSM, Team “Pathogènes Hydriques Santé et Environnements”, Unit of Bacteriology, Faculté de Pharmacie, Montpellier, France; 3 Department of internal medicine and geriatrics, HUG de Genève, Genèva, Switzerland; 4 Department of Clinical microbiology, CHU de Nice, Nice, France; 5 Inserm U1065, Centre Méditerranéen de Médecine Moléculaire, Team « Microbial toxins in host pathogen interactions », Nice, France; 6 Université de Nice-Sophia Antipolis, Faculté de Médecine, Nice, France

**Keywords:** Leech therapy, Healthcare-associated infections, Practices of leech management, National survey

## Abstract

**Background:**

Leech therapy in plastic/reconstructive microsurgery significantly improves a successful outcome of flap salvage but the drawback is a risk of severe infection that results in a drop of the salvage rates from 70-80% to below 30%. We report the results of a national survey conducted in all the French university hospitals to assess the current extent of use of leech for medical practices in the hospital and to investigate maintenance, delivery practices and prevention of the risk of infection.

**Methods:**

Data concerning conditions of storage, leech external decontamination, microbiological controls, mode of delivery and antibiotic prophylaxis were collected from all the French university hospitals in practicing leech therapy, on the basis of a standardized questionnaire.

**Results:**

Twenty-eight of the 32 centers contacted filled the questionnaire, among which 23 practiced leech therapy, mostly with a centralized storage in the pharmacy; 39.1% of the centers declared to perform leech external decontamination and only 2 centers recurrent microbiological controls of the water storage. Leech delivery was mostly nominally performed (56.5%), but traceability of the leech batch number was achieved in only 39.1% of the cases. Only 5 centers declared that a protocol of antibiotic prophylaxis was systematically administered during leech therapy: either quinolone (2), sulfamethoxazole/trimethoprim (2) or amoxicillin/clavulanic acid (1).

**Conclusions:**

Measures to prevent infectious complications before application to patient have to be better applied and guidelines of good practices are necessary.

## Background

During the two latest decades, the use of leeches for medicinal purposes has increased because of their effectiveness in enhancing venous outflow by removing the stagnant blood [1]. Leech therapy is widely used in plastic and reconstructive microsurgery to aid salvage of failing flaps, replanted digits, ears or lips [2–5]. It significantly improves a successful outcome but the drawback is a risk of infectious complication that results in a drop of the salvage rates from 70-80% to below 30% [1]. Incidence rates of infection have been reported between 4.1% and 36.2% [6, 7], and the vast majority of infections are mostly caused by aeromonads [8]. These ubiquitous opportunistic bacteria are mainly found in aquatic environments and are present in the digestive tract of leeches as an obligate symbiont [9]. Infection is usually severe (e.g., loss of flap) so that animals are decontaminated before use to reduce risk of infection, and antibiotic prophylactic treatment (ATBP) is widely recommended during leech application [7, 10, 11].

Ricarimpex SAS (Eysines, France), the exclusive supplier of leeches in France and international leader (FDA approved), clearly recommends conditions of storage, but neither the conditions of leech decontamination, the need to perform environmental surveillance cultures, nor the type of ATBP regimen are advised. In addition, no clear guideline of good practices for leech management before use exists in France. Guidelines describing procedures of procurement and storage, transport, application, removal and discarding of leeches and the use of antibiotics during leech therapy are available in several countries. For example, American guidelines describe procedure of maintenance of leeches specifying not to use tap water to the storage, or English and Australian guidelines which recommend to administrate an antimicrobial cover during hirudotherapy [12–14].

To assess the current extent of use of leech for medical practices in the hospital and to investigate maintenance, delivery practices and infection control measures, we report the findings of a national survey conducted in all the French university hospitals.

## Methods

A questionnaire was sent by email in August 2015 to the pharmacy departments of all the institutions belonging to the “CHU network”, a network that includes all the 32 French university hospitals and related centers. A second email was sent to non-responders in November 2015. Responders were asked to complete the questionnaire and to return it by email. Data were analyzed descriptively after collection. The following data were collected, on the basis of a standardized questionnaire, from the pharmacist in charge of medicinal leech management:

- Conditions of storage: type of containers, type of water, temperature of storage and frequency of tank water change.
- Leech external decontamination: frequency of decontamination, type of disinfectant used and description of the modalities of decontamination.
- Microbiological control of the leeches or of the storage water and description of the modalities of controls.
- Leech delivery: by patient or overall by wards and traceability of the batch number.
- Antibiotic prophylaxis: antibiotic used, length of therapy and existence of local recommendations.

## Results

**Fig. 1:**
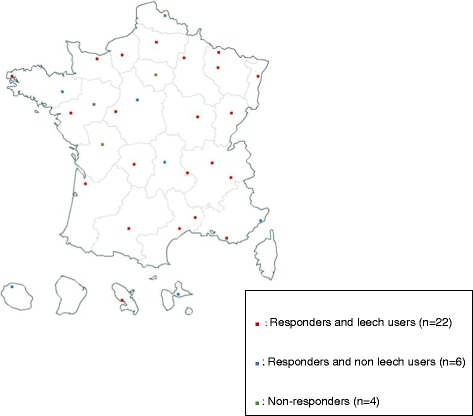
Regional repartition of the French participating centers (*n* = 32)

**Fig. 2:**
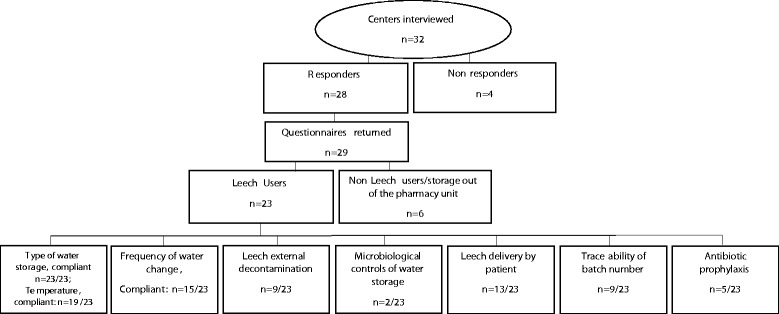
Flow-chart of the study showing the distribution of the responders and non-responders French centers

Overall, information was obtained from 28 out of the 32 institutions interviewed (87.5%), including our center (Fig. 1). Two questionnaires were returned by one hospital because it was in charge of 2 centers of microsurgery with differing practices in the leech management. Five of the 28 responders (17.9%) stated that they did not use leeches in their hospital, and one center was unaware of the protocol in use because leeches were not delivered by the pharmacy but were directly stored in the surgery unit. A flow-chart of the study is presented in Fig. 2.

In 2015, French centers ordered an annual average of 485 leeches (minimum: 30; maximum 1300) per center (information obtained from 52% of the centers using leeches).

**Fig. 3:**
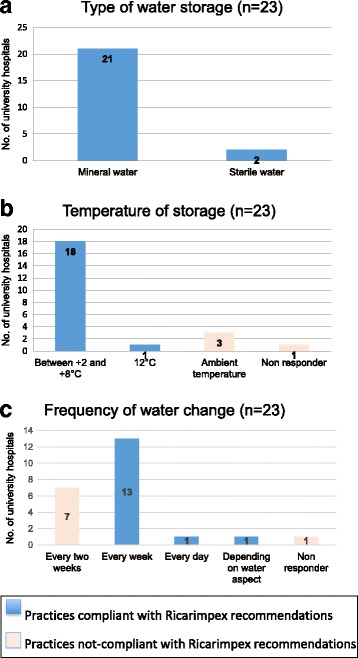
Practices of leech storage within Pharmacy departments of French university hospitals using leeches. **a**, type of water storage; **b**, temperature of storage; **c**, frequency of tank water change (*n* = 23)

Ricarimpex recommendations, displayed in Table 1, are warranted by the leech susceptibility to external conditions as light, temperature, and varying substances present in aquatic environments, and should be applied as a minimum standard. All the responders declared using a type of water in accordance with Ricarimpex recommendations and 19 centers (82.6%) used a temperature of water storage compliant to supplier’s guidelines (Fig. 3a and b). Tank water change was achieved at a frequency that was in accordance with the supplier guideline in only 65% of the cases (15/23) and 7 centers (30.5%) changed water less frequently (every two weeks); one center did not respond (Fig. 3c).

**Table 1:** Ricarimpex recommendations for leech storage

- Sterile jar (or clean) closed with a sterile gauze	
- Water storage: either mineral water, mountain spring water or distilled water with Hirudo salt	
- Change water at least once a week or in case of troubled water	
- Temperature of storage between + 4 and + 20 °C in a dark place	
- Conserve the batch number and do not mix leeches from different batch numbers

Nine centers (39.1%) among the 23 that used medicinal leeches declared to perform leech external decontamination. Overall, 7 centers practiced decontamination with chlorhexidine and 2 centers with gentamicin. The decontamination was performed at time of leech delivery for 6 centers, at time of leech application for 1 center, and systematically during all the storage period for 2 centers, at a frequency of one decontamination every week and every 2 weeks, respectively.

Only 2 pharmacy departments declared to perform recurrent microbiological controls of the water storage: one center performed water control just after leech reception or when the visual quality of the water was deteriorated, and the other center performed water controls every month.

Leech delivery was mainly performed by patient (13/23) and traceability of the batch number was achieved in 39.1% of the cases (9/23).

Five centers (21.7%) declared that a protocol of ATBP was systematically administered during leech therapy, and the drug administered was quinolone (2 centers), sulfamethoxazole/trimethoprim (2 centers) and amoxicillin/clavulanic acid (1 center). In these 5 centers, the ATBP regimen was defined by the surgeon for 2 centers; by the local antibiotic committee for 2 others centers, and one center declared to follow the recommendations of the supplier despite no guideline on ATBP from Ricarimpex exists yet. In these 4 centers, the ATBP was maintained during the total duration of leech therapy, and in one case with an average duration of 10 to 15 days. Three other centers reported to use ATBP, although not systematically, and decision to administer ATBP depended on the leech therapy indication or on the health status of the patient (e.g., immunocompromized patient). Finally, 9 (39.1%) centers did not use ATBP, and 6 (26.1%) additional centers did not respond to the question.

## Discussion

This survey provides an overview of the current French hospital practices for managing leech before use on plastic surgery patients. We focused on university hospitals because this is where reconstructive surgery is overall concentrated, although we may have missed few other centers practicing leech therapy. Despite this limitation, we present almost exhaustive results reflecting French hospital practices for leech maintenance and this study is comparable with the national survey of Taneja et al. conducted in United Kingdom [15]. These authors studied practices of use and application of leeches in surgery wards, while we evaluated practices of maintenance within pharmacy departments.

### Leech storage before use and delivery

This national survey showed that nearly 70% of the French University Hospitals use leech therapy, confirming a wide use of this recent practice. Mostly, leeches are stored in the pharmacy department, which enables a better control of leech management prior to delivery and to application. This includes a centralized maintenance, a controlled traceability and an easier surveillance in case of nosocomial infection related to leech therapy. Sartor et al. suggested that storage conditions and quality of water could increase the risk of contamination related to the leeches. When leeches, delivered initially by the pharmacy, were stored in the hand surgery unit in an aquarium that was not regularly decontaminated, infections were twice as common (4.1% vs 2.4% respectively) as in the reconstructive units that received leeches directly from the pharmacy before use. This observation led the authors to recommend maintaining leeches at a central site, with strict protocols of cleaning and disinfection of the aquariums [6].

From our experience, a centralized leech storage and delivery is obviously the best practice to recommend, in agreement with American, English and German Guidelines [12–14].

Leech delivery was registered in only 56.5% of the cases on a database in the pharmacy and was nominative per patient, while 7 centers practiced a global delivery to the medical or surgical units. Despite the lack of guidelines, we consider that leech should be nominally delivered and batch number recorded, in order to allow optimal investigation in case of healthcare-associated infection related to leech therapy. Overall and in a safer way, the delivery must be limited to a single use only and then discarded to avoid any risk of infection transmission between patients, as suggested in the protocol for the use of leeches by some authors [16, 17].

### Microbiological controls prior leech use

There is no guideline concerning either the need or the procedure of an environmental surveillance culture of leech tank water. Wilmer et al. advocated microbiological surveillance in routine to establish the ecology of water, arguing that identifying resistant organisms would allow clinicians to adjust ATBP regimens [18]. With the aim to limit serious wound infections, Verriere et al. are also in favor to perform microbiological controls of the water tank, allowing to detect resistant strains, to discard the corresponding leeches and to select an appropriate ATBP [19]. However, the finding of only susceptible isolates does not preclude the presence of resistant isolates that would be present at low density in the leech crop and would remained undetected. The level of evidence is to date too poor to recommend to implement such policy. It is not established that either the results of microbiological analysis of water tank or leech microbiota would accurately predict the bacteria involved in infection following leech therapy. Indeed, data suggest that the leech gut microbiota is more complex than initially thought [20] and includes many distinct *Aeromonas* strains. In addition, host-leech-microbiota is a complex biological system that must not be ignored in which the dominant strain(s) in the leech gut or in the tank water is not always the dominant strain in the water tank or the pathogenic strain in the patient. Until a greater body of evidence including a cost effectiveness analysis, it is tricky to recommend a systematic microbiological control to predict leech safety or to adapt prophylactic antimicrobial treatment.

### Antibiotic prophylactic treatment

Despite the numerous publications widely showing the interest of ATBP during leech therapy [15, 19, 21, 22], ATBP was prescribed in only 40% of the French hospitals interviewed. Overall, this result, together with the worrying percentage of no response (6 centers, 26%), suggests that the frequency and severity of this nosocomial infection is underrated by clinicians, as demonstrated elsewhere [21]. Most of authors suggest to start ATBP prior or when starting the application of leeches on patient, to administer antibiotics during all the duration of the leech therapy, and to end it 24 h after the end of leech application [7, 16]. Lineaweaver et al. suggested to extend ATBP until cicatrization has occurred [10]. Consensual guidelines on ATBP associated with the use of leeches are needed. Antibiotics the most frequently encountered in protocols of ATBP associated with leech therapy are fluoroquinolones, sulfamethoxazole/trimethoprim and third generation cephalosporins [7, 21], although several authors have reported since 2012 the emergence of *Aeromonas* resistant strains, particularly to ciprofloxacin [16, 23–25]. Amoxicillin/Clavulanic acid should be avoided because aeromonads are virtually all resistant to this antimicrobial agent because oxacillinase and/or cephalosporinase is expressed by the vast majority of aeromonads [26–28]. In our center, all prescribers of medicinal leeches are informed on the risk of infection related to leech therapy, and are advised on prophylactic treatment by a letter systematically accompanying leech delivery.

### Other approaches for controlling the risk of infection

In order to reduce the risk of infection related to leech therapy, alternatives to ATBP have been described. Mackay et al. tried to sterilize the leech gut of pathogens by immersing leeches in an antibiotic solution during 12 h [29]. Mumcuoglu et al. proposed to eliminate *Aeromonas* sp. of the leech digestive tract by feeding them with an arginine solution supplemented with ciprofloxacin. All these attempts were unsuccessful [30]. Such approach should be strongly discouraged because of the risk of selecting resistant mutants (e.g., to ciprofloxacin). Alternatively, further research is required to identify new strategies for removing the aeromonads or at least drastically decreasing their density from leech gut, but the objective is obviously difficult to achieve because of the obligate symbiosis between leech and aeromonads.

We show here that 39% of the French hospitals perform an external decontamination, among which 77.7% used chlorhexidine. Bauters et al. described similar practices of decontamination in a Belgian hospital, treating leeches with chlorhexidine 0.02% during 15 s, followed by successive rinses with sterile water [31]. Comprehensively, the level of evidence is rather low to identify the best practice, and there is consequently no consensus on the optimal method to use. Further study that improves knowledge on this point should be welcomed, but the principle of an external decontamination ensuring a nearly germ-free state during few hours and preserving the suckle capability of the leech should reasonably be recommended [32]. Indeed, flaps or (re) implanted tissues are associated with a local immunosuppression, and the wound caused by the jaws of the leech offers a cutaneous entry point by contiguity. In a general way, a consensual approach is needed with at least European or even international Guidelines [8].

### Legal status of medicinal leeches

Leech is a strikingly unparalleled product in healthcare practice, so that the legal status was not, until recently, clearly established. Many countries experienced the need to regulate for health safety purposes, and this resulted in diverse status: the French National Agency for Medicines and Health Products Safety (ANSM) conferred to medicinal leeches the status of therapeutic aid; the United States Food and Drug Administration (FDA) the status of a medical device, approved in 2004 [33]; in the UK, the Medicines and Healthcare Products Regulatory Agency (MHRA) conferred the status of a medicinal product when leeches are used with an obvious medical purpose [34], and the German Federal Institute for Drugs and Medical devices (BfArM) conferred to starved leeches, the status of drug since 2008 [14]. German recommend specifically a quarantine storage to starve leeches during at least 32 weeks after the last feed. Although the level of scientific evidence is not established for preventing the risk of infection to *Aeromonas*, it likely controls potential viral risks.

## Conclusion

In conclusion, leech therapy is associated with a high risk of severe infection, currently impossible to eliminate. Further research is necessary to understand how to eliminate this risk, and will likely require a deep understanding of a complex pathophysiology involving host, leech, and leech microbiota. Meanwhile, measures known to control this risk must clearly be protocoled and actually implemented. The wide variation of practices observed here highlights that they are insufficiently fulfilled and advocates for establishing guidelines. It is prudent that the practices discussed above be strictly applied: supplier’s guideline and quality of leech storage, leech decontamination and antibiotic prophylactic treatment. Failure to comply with these measures increases the risk of infection. On the opposite, microbiological control of leech tank water should not be promoted until more convincing data are provided, as it likely constitutes a false security.

At last, a multidisciplinary collaboration between the microbiology laboratory, the infectious disease unit, the pharmacy and the infection control departments is advised in every center practicing leech therapy to report, monitor nosocomial outcomes and to maintain the risk of infection as low as possible.

## Abbreviation

ATBP: Antibiotic prophylactic treatment
